# Co-Treatment with the Epigenetic Drug, 3-Deazaneplanocin A (DZNep) and Cisplatin after DZNep Priming Enhances the Response to Platinum-Based Therapy in Chondrosarcomas

**DOI:** 10.3390/cancers13184648

**Published:** 2021-09-16

**Authors:** Eva Lhuissier, Juliette Aury-Landas, Marion Lenté, Karim Boumediene, Catherine Baugé

**Affiliations:** EA7451 BioConnecT, Normandie University, UNICAEN, 14032 Caen, France; lhuissier.eva@gmail.com (E.L.); juliette.aury-landas@unicaen.fr (J.A.-L.); marion.lente11@gmail.com (M.L.); karim.boumediene@unicaen.fr (K.B.)

**Keywords:** apoptosis, bone tumors, chondrosarcoma, cell death, xenograft, cisplatin, cancer, chemotherapy, methyltransferase, epidrugs

## Abstract

**Simple Summary:**

Chondrosarcoma is a rare bone tumor characterized by the secretion of a cartilage-like extracellular matrix. Its treatment poses major challenges, since chondrosarcoma is resistant to chemotherapy and radiotherapy. Consequently, chondrosarcoma treatment has been limited over the past 30 years, and consists in the surgical resection of the tumor. Increasing evidence suggests that future cancer therapies will be enhanced by the combination of epigenetic and conventional antitumor agents, leading to further investigations to combine 3-Deazaneplanocin A (DZNep), an epigenetic drug, with existing antitumoral agents. We show by in vitro and in vivo experiments that an optimised DZNep/cisplatin combination reduces chondrosarcoma viability and induces apoptosis more effectively than each of the drugs alone. These results demonstrate the potential use of this epigenetic-chemotherapeutic combination approach for further studies and management of chondrosarcoma treatment.

**Abstract:**

Background: We have previously shown that 3-Deazaneplanocin A (DZNep) induces apoptosis in chondrosarcomas. Herein, we tested whether the combination of this epigenetic drug to a standard anticancer therapy may enhance the response to each drug in these bone tumors. Methods: Two chondrosarcoma cell lines (SW1353 and JJ012) were cultured in the presence of DZNep and/or cisplatin. Cell growth was evaluated by counting viable cells, and apoptosis was determined by Apo2.7 expression by flow cytometry. In vivo, the antitumoral effect of the DZNep/cisplatin combination was assessed through measurements of tumor volume of JJ012 xenografts in nude mice. Results: In vitro, the DZNep/cisplatin combination reduced cell survival and increased apoptosis compared to each drug alone in chondrosarcomas, but not in normal cells (chondrocytes). This enhancement of the antitumoral effect of the DZNep/cisplatin combination required a priming incubation with DZNep before the co-treatment with DZNep/cisplatin. Furthermore, in the chondrosarcoma xenograft mice model, the combination of both drugs more strongly reduced tumor growth and induced more apoptosis in tumoral cells than each of the drugs alone. Conclusion: Our results show that DZNep exposure can presensitize chondrosarcoma cells to a standard anticancer drug, emphasizing the promising clinical utilities of epigenetic-chemotherapeutic drug combinations in the future treatment of chondrosarcomas.

## 1. Introduction

Chondrosarcoma is a rare bone tumor characterized by the secretion of a cartilage-like extracellular matrix. Its treatment poses major challenges, since chondrosarcoma is resistant to chemotherapy and radiotherapy. Consequently, chondrosarcoma treatment has been limited over the past 30 years, and consists in the surgical resection of the tumor [1,2].

Recently, 3-Deazaneplanocin A (DZNep), a carbocyclic adenosine analog, has been shown to be able to induce apoptosis in chondrosarcomas in vivo and in vitro [3,4,5]. DZNep is an inhibitor of S-adenosylhomocysteine hydrolase, leading to the cellular accumulation of S-adenosylhomocysteine and the inhibition of S-adenosyl-methionine-dependent methyltransferases, particularly histone methyltransferases [6,7]. It induces apoptotic cell death in cancer cells, but not in normal cells [8,9,10]. Its anti-tumoral activity has also been observed in vivo in numerous cancers, including embryonal rhabdomyosarcoma [11], tongue cancer [12], prostate cancer [13], renal cell carcinoma [14], or lung cancer [15]. In chondrosarcomas, DZNep induces apoptosis in vitro [4] and slows down tumor growth in vivo [3,5]. Interestingly, this epigenetic drug appears to be relatively safe [16,17].

Increasing evidence suggests that future cancer therapies will be enhanced by the combination of epigenetic and conventional antitumor agents, leading to further investigations to combine DZNep with existing antitumoral agents. For instance, histone methylation reversal by DZNep presensitizes pancreatic cancer cells to gemcitabine [18]. Also, DZNep treatment potentiates the antitumor effect of cisplatin in non-small cell lung cancer cells, and protects against its nephrotoxicity [19]. Thus, an epigenetic-chemotherapeutic drug combination might provide an efficient strategy therapy for cancers [20].

Therefore, our study attempts to increase the efficiency of antitumor drugs in chondrosarcomas by combining DZNep with standard chemotherapy agents, such as platinum compounds. We show by in vitro and in vivo experiments that an optimised DZNep/cisplatin combination reduces chondrosarcoma viability and induces apoptosis more effectively than each of the drugs alone.

## 2. Materials and Methods

### 2.1. Drugs

DZNep-HCl was provided by Tocris (Lille, France). Cisplatin was provided by Sigma (St Quentin Fallavier, France).

### 2.2. Cell Culture

Human chondrosarcoma cell lines SW1353 and JJ012 were purchased from the American Type Culture Collection (ATCC, Manassas, VA, USA), and kindly provided by Dr. Joel A. Block (Rush University Medical Center) [21,22], respectively. The identity of cell lines was confirmed using STR profiling with the GenePrint 10 System (Promega, Charbonnières-les-Bains, France). Chondrocytes were isolated and cultured, as previously described, from normal articular cartilage [23], after obtaining signed agreement from patients, according to local legislation and in accordance with the recommendation of local ethic committee (Comité de Protection des Personnes Nord Ouest III).

Normal and tumoral cells were cultured in Dulbecco’s Modified Eagle Medium (DMEM) supplemented with 10% fetal bovine serum (FBS) (Invitrogen, Cergy-Pontoise, France) and antibiotics (penicillin/streptomycin), and incubated at 37 °C in a humidified atmosphere containing 5% CO_2_. Cell cultures were regularly tested by PCR for mycoplasma contamination.

### 2.3. Cell Growth Experiments

Cells were seeded at 15,000 cells/cm^2^. The day after, they were treated with drugs. At the end of incubation, viable cells were counted using Countess II (Life Technologies, Illkirch, France) after trypan blue exclusion. Each count was performed twice, and at least three independent experiments were done.

### 2.4. Apoptosis Assay

Apoptosis was assayed as previously described [3]. Briefly, cells were immunostained with phycoerythrin (PE)-conjugated antibody directed against Apo2.7 (clone 2.7 7A6) according to the manufacturer’s condition (Beckman Coulter, Villepinte, France). Fluorescence was measured using the Gallios flow cytometer (Beckman Coulter, Villepinte, France) at the FACS facility (SFR 146 ICORE, Caen, France). At least 10,000 events were analysed in each sample. Three independent experiments were performed.

### 2.5. Animals

Animal experimental procedures were performed according to local legislation, after approbation by the ethics committee (Comité d’Ethique de Normandie en Matière d’Expérimentation Animale, agreement #03968.01). Mice were provided by Charles River (L’Arbresle, France) and then kept in the animal facility (Centre Universitaire de Ressources Biologiques, Caen, France). The mice were maintained in standard polypropylene cages (37 × 23.5 × 18 cm, Charles River, 5–6 mice per cage) in a temperature- and humidity-controlled room, and had free access to water and food. The animal investigations were performed under the current European directive (2010/63/EU) as incorporated in national legislation (Decree 87/848). Each animal was humanely handled throughout the experiment, in accordance with internationally accepted ethical principles for laboratory animal use and care, and all efforts were made to minimize animal suffering. Euthanasia was performed using isoflurane inhalation.

### 2.6. Xenograft of Chondrosarcomas in Nude Mice

Twenty-five *SWISS nude* mice (6 weeks old, male, weight average = 32.23 g (28.50–36.5)) were xenografted subcutaneously with 10^6^ JJ012 cells (suspended in 100 μL of matrigel) on their back, as previously described [3], and separated in four groups: vehicle (*n* = 4, weight average = 31.6 g (29.5–33.8)); DZNep alone (*n* = 7, weight average = 31.04 g (28.70–33.40)); cisplatin alone (*n* = 7, weight average = 33.44 g (29.50–36.50)); and DZNep and cisplatin in combination (*n* = 7, weight average = 32.56 g (28.50–36.40)). When the tumors became palpable (about 100 mm^3^), vehicle or DZNep was administered intraperitoneally three times per week at 2 mg/kg for 4 weeks (“priming” stage). Then, cisplatin was intraperitoneally injected alone or in combination with DZNep three times per week at 2 mg/kg for 3 weeks (“treatment” stage). During experiments, tumors were measured two times per week with a caliper, and tumoral volume was calculated by the following equation: (L × w^2^)/2 (with L corresponding to length and w to width).

### 2.7. Protein Extraction and Western Blot

Tumors were crushed with potter, and lysed in a RIPA lysis buffer (Tris-HCl 50 mM pH 7.5; IGEPAL 1%; NaCl 150 mM; EGTA 1 mM; NaF 1 mM) supplemented with phosphatase inhibitors (NA_3_VO_4_ 10 µL/mL) and protease inhibitors (leupeptin 1 µL/mL; aprotinin 1 µL/mL; pepstatin 1 µL/mL; and phenylmethylsulfonyl fluoride 4 µL/mL). Protein extracts were migrated in SDS-PAGE, and transferred to polyvinylidene difluoride (PVDF) membranes (Bio-Rad, Marnes-la-Coquette, France). After probing with primary antibodies, the membranes were incubated with horseradish peroxidase-conjugated secondary antibodies, and signals visualized by Western Lightning^®^ Plus-ECL (Perkin Elmer, Villebon S/Yvette, France). Antibodies specific for PARP (#5246), H3K4me3 (#9727), H3K9me3 (#13969), H3K27me3 (#9733), H3K36me3 (4909), H3K79me3 (#4260), H4K20me3 (#5737), and H3 (#4499) were obtained from Cell Signalling (Danvers, MA, USA), and actin (sc47778) was provided by Santa Cruz (Heidelberg, Germany). The whole Western blot figures can be found in File S1.

### 2.8. Statistical Analysis

All data are expressed as mean ± SEM. For in vitro experiments, at least three different experiments were performed for each condition. Statistical significances were calculated with Student’s-*t*-test. For in vivo experiments, global analysis of tumor volumes was performed using a two-way ANOVA with repeated measurements, followed by post-hoc Tukey multiple comparison. All data are expressed as mean ± SEM, and *p*-values < 0.05 were considered as statistically significant.

## 3. Results

### 3.1. DZNep Reduces Histone Methylation in Chondrosarcomas

**Figure 1:**
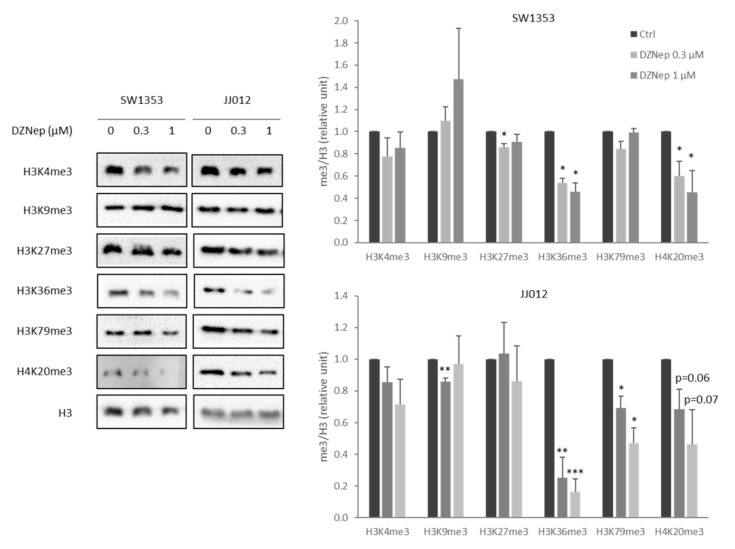
DZNep treatment reduces histone trimethylation level in chondrosarcomas. SW1353 or JJ012 chondrosarcomas were treated with increasing doses of DZNep (0.3 µM and 1 µM) for two days. Then, proteins were extracted, and histone trimethylation levels were analyzed by western blot. H3 was used as the protein loading control. Representative figures of three independent experiments are shown. Histograms represent the relative quantification of me3/H3 signals. Data are expressed as means +SEM (*n* = 3). *: *p*-value < 0.05; **: *p*-value < 0.01; ***: *p*-value < 0.001.

In this study, we used two chondrosarcoma cell lines: SW1353, a cell line that is not very sensitive to cisplatin, and JJ012, a cell line that is more sensitive to cisplatin. We first evaluated whether DZNep is able to modify the histone methylation profile in both chondrosarcoma cell lines. After 48 h, DZNep strongly reduced the trimethylation of histones H3 at lysine 36 (H3K36me3) and H4 at lysine 20 (H4K20me3) in a dose-dependent manner. It also decreased H3K79me3 and H3K4me3 in the JJ012 line (Figure 1). These data confirm that DZNep is an epigenetic drug, which regulates histone methylations.

### 3.2. Co-Treatment DZNep/Cisplatin Is Not Sufficient to Reduce Survival Compared to Cisplatin Treatment

**Figure 2:**
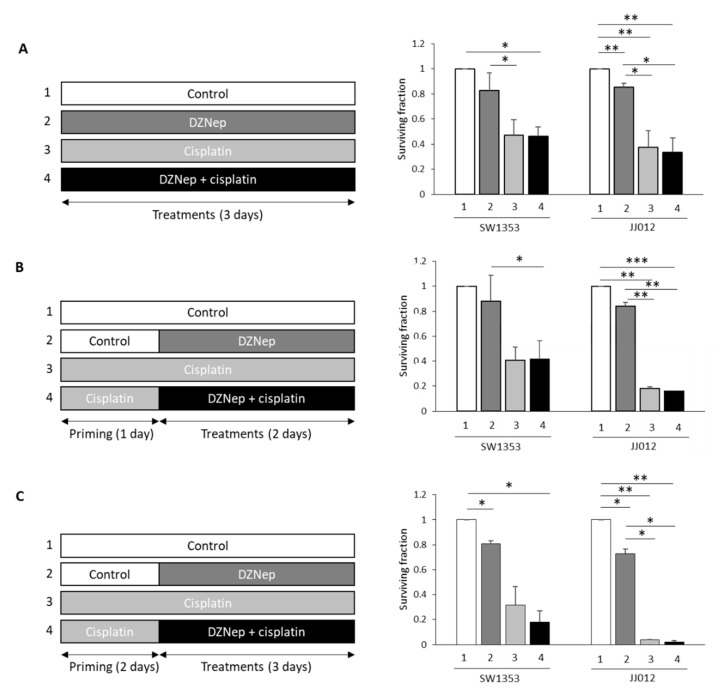
Direct co-treatment of DZNep/cisplatin or cisplatin priming did not affect cell survival. SW1353 or JJ012 chondrosarcoma cells were treated with DZNep (0.3µM) and cisplatin (5µM). (**A**) Co-treatment Cisplatin/DZNep for three days. (**B**) Primed with cisplatin (one day), following co-treatment cisplatin/DZNep (two days). (**C**) Primed with cisplatin (two days) following by co-treatment of cisplatin/DZNep (three days). At the end of the experiments, viable adherent cells were counted. Cell survival was normalized to untreated cells. *: *p*-value < 0.05; **: *p*-value < 0.01; ***: *p*-value < 0.001.

Next, we compared the response of chondrosarcomas to cisplatin and DZNep alone or in combination. We used drugs at concentrations that are close to the IC50 values in the less-sensitive-to-cisplatin cell line (SW1353), and that did not induce apoptosis in normal cells (chondrocytes). We evaluated whether direct DZNep/cisplatin co-treatment (Figure 2A), or cisplatin priming followed by DZNep/cisplatin co-incubation (Figure 2B,C), are able to reduce chondrosarcoma survival. None of the combinations were able to induce a reduction of chondrosarcoma survival compared to cisplatin alone.

### 3.3. Co-Treatment with DZNep and Cisplatin after DZNep Priming Enhances the Sensibility to Cisplatin in Chondrosarcomas

**Figure 3:**
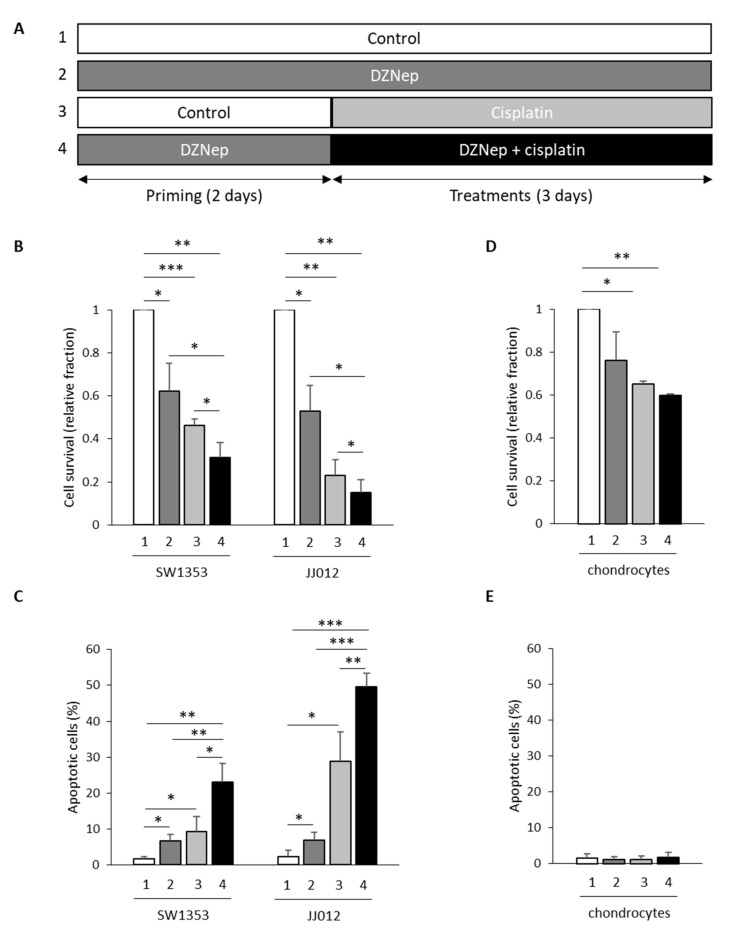
Co-treatment with DZNep and cisplatin after DZNep priming increased the effect of cisplatin on cell survival and on apoptosis in chondrosarcoma cells, but not in normal cells. SW1353 or JJ012 chondrosarcomas, and normal cells (chondrocytes) were primed or not with DZNep (0.3 µM) for two days and then treated with DZNep (0.3 µM) and/or with cisplatin (5 µM) for three days. (**A**) Experimental design. (**B**,**D**) Viable adherent cells were counted at the end of treatments. Graphs represent cell survival normalized to untreated cells. Data are expressed as means ± SEM. (**C**,**E**) Apoptosis was assayed by staining cells with Apo 2.7 antibody coupled with phycoerythrin. Histograms represents the percentage of apoptotic cells. Data are expressed as means ± SEM. *: *p*-value < 0.05; **: *p*-value < 0.01; ***: *p*-value < 0.001.

Next, we evaluated whether DZNep priming followed by DZNep/cisplatin co-treatment enhances the sensibility to cisplatin alone (Figure 3A). We compared the response of chondrosarcomas to cisplatin and DZNep alone or in combination (Figure 2A). We used drugs at concentrations that are close to the IC50 values in the less-sensitive-to-cisplatin cell line (SW1353), and that did not induce apoptosis in normal cells (chondrocytes). We showed that DZNep and cisplatin significantly reduced the viability of chondrosarcomas. Interestingly, this reduction of cell survival was significantly greater when chondrosarcomas were treated by the combination of DZNep/cisplatin after DZNep priming (corresponding to a priming period with DZNep 0.3 µM for two days, followed by a treatment period for three days with a combination of DZNep 0.3 µM and cisplatin 5 µM), compared to treatments with each drug alone (Figure 3B).

We further analyzed the effect of drugs alone or in combination on apoptosis. Both treatments with DZNep (0.3 µM for five days) or cisplatin (5 µM for three days) moderately induced apoptosis. In contrast, two days of DZNep priming followed by treatment for three days with both drugs significantly increased apoptosis (Figure 3C).

### 3.4. Co-Treatment with DZNep and Cisplatin after DZNep Priming Does Not Enhance Chondrocyte Sensibility to Cisplatin

Furthermore, we investigated whether treatments with DZNep and cisplatin alone or in combination are toxic in normal cartilage cells, e.g., chondrocytes. Even if the combination of DZNep and cisplatin reduced the chondrocyte cell number (−40%) after five days compared to untreated cells (Figure 3D), it did not induce apoptosis in these cells (Figure 3E), suggesting that it only reduced cell proliferation without leading to cell death in chondrocytes.

### 3.5. Peritoneal Injection of DZNep and Cisplatin Reduces Tumor Growth in Xenograft Mice

**Figure 4:**
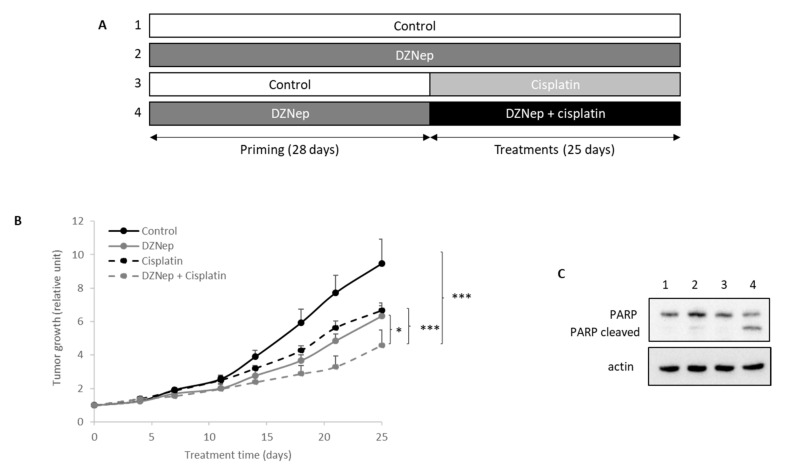
Peritoneal injections of DZNep and cisplatin reduced tumoral growth of chondrosarcoma in xenograft nude mice. The JJ012 chondrosarcoma cell line was subcutaneously injected in 25 nude mice. When tumors were implanted, DZNep (2 mg/kg) was intraperitoneally injected three times per week. After four weeks of DZNep priming, mice were treated with DZNep and/or cisplatin (2 mg/kg i.p) three times per week for three weeks. (**A**) Experimental design. (**B**) Tumors were measured regularly by a caliper and tumoral volume was calculated. Values represent means ± SEM. *: *p*-value < 0.05; ***: *p*-value < 0.001. (**C**) At the end of experiment, proteins were extracted from tumors, and PARP cleavage was analyzed by western blot.

To extend this analysis, we performed in vivo experiments using nude mice bearing xenografted JJ012 tumors (chondrosarcomas cell lines with better capacity of implantation than SW1353). As shown in Figure 4, DZNep priming followed by DZNep/cisplatin co-treatment significantly slowed down tumor growth compared to treatment with a single drug (see Table S1 for raw values). No change in the weight of the mice treated with cisplatin, DZNep, or both drugs was observed.

Moreover, apoptosis in untreated or DZNep-treated tumors was also analyzed. The data revealed that the combination of cisplatin with DZNep increased apoptosis as evidenced by the PARP cleavage (Figure 4C).

## 4. Discussion

Chondrosarcoma treatment remains an important issue for clinicians. Indeed, this bone tumor is resistant to conventional radio- and chemotherapy, and its resection is the usual treatment. In this context, there is an important need to identify novel drugs able to treat chondrosarcoma. The present study demonstrates that the combination of 3-deazaneplanocin and cisplatin exhibits strong pro-apoptotic effects on chondrosarcomas in vitro and in vivo, superior to the effects of the drugs alone.

We previously showed that DZNep, an inhibitor of SAH known to reduce histone methyltransferase activity, induces apoptosis in vitro and in vivo in chondrosarcomas [3,4,5], without inducing deleterious effects on the majority of tissues, nor on cognitive functions in mice [16]. In the present study, we show that co-incubation with DZNep and cisplatin after DZNep priming enhances apoptosis compared to treatment with the drugs alone. This is in accordance with the literature. Indeed, in other cancer cells, such as cholangiocarcinoma, pancreatic cancer, or rhabdoid tumor cells, the combination of DZNep with standard anticancer agents (gemcitabine, etoposide, or doxorubicin) induces a synergistic inhibition of cell proliferation and significantly increases apoptosis compared to the drugs alone [24,25,26].

Furthermore, and interestingly, DZNep and cisplatin alone as well as in combination did not induce apoptosis in normal chondrocytes. Similarly, DZNep/gemcitabine co-incubation does not enhance drug toxicity in normal pancreatic cells [18]. Additionally, we observed no change in the weight of the mice treated with cisplatin, DZNep, or both drugs, suggesting that, at the doses used in this study, these drugs do not have major toxic effects in vivo.

Intriguingly, this enhancement of the cytotoxic effect by combination of DZNep/cisplatin was only observed when cells were primed with DZNep, but not for a direct co-treatment. This temporal difference was also observed by Hung and collaborators, which showed that DZNep priming has superior cytotoxicity and synergy with gemcitabine than the co-exposure of both drugs in pancreatic cancer cells [18].

The mechanism by which DZNep potentialize chemotherapeutic drugs is still unclear. Some have proposed that DZNep, by reducing EZH2 activity and H3K27 trimethylation, a mark of heterochromatin, may induce a loss of chromatin compaction, which could favour DNA damage induced by conventional cytotoxic drugs (such as platinum compounds) and consequently reduce the survival of cancer cells. However, in some chondrosarcomas (as JJ012), DZNep did not reduce H3K27me3. However, DZNep is known to reduce global histone methylation, and herein, we show that this drug significantly reduces the trimethylation of H3K36, H4K20, H3K79, and H3K4, confirming that DZNep acts though epigenetic mechanisms.

## 5. Conclusions

We have developed a procedure to enhance the apoptosis induced by cisplatin and DZNep in human chondrosarcomas. While cisplatin alone only produces modest effects in theses bone tumors, DZNep priming followed by DZNep/cisplatin co-treatment enhances the overall pro-apoptotic efficacy of each of the drugs in chondrosarcoma cells, while preserving normal chondrocytes. These results demonstrate the potential use of this epigenetic-chemotherapeutic combination approach for further studies and management of chondrosarcoma treatment.

## Data Availability

The data presented in this study are available in supplemental data, or available on request from the corresponding author.

## Supplementary Materials

The following are available online at https://www.mdpi.com/article/10.3390/cancers13184648/s1, Table S1: Tumoral volumes of chondrosarcoma xenografts during priming and treatment periods. File S1: the whole western blot figures.

## References

[1] Giuffrida A.Y., Burgueno J.E., Koniaris L.G., Gutierrez J.C., Duncan R., Scully S.P. (2009). Chondrosarcoma in the United States (1973 to 2003): An analysis of 2890 cases from the SEER database. J. Bone Jt. Surg. Am..

[2] Leddy L.R., Holmes R.E. (2014). Chondrosarcoma of bone. Cancer Treat. Res..

[3] Lhuissier E., Bazille C., Aury-Landas J., Girard N., Pontin J., Boittin M., Boumediene K., Baugé C. (2017). Identification of an easy to use 3D culture model to investigate invasion and anticancer drug response in chondrosarcomas. BMC Cancer.

[4] Girard N., Bazille C., Lhuissier E., Benateau H., Llombart-Bosch A., Boumediene K., Bauge C. (2014). 3-Deazaneplanocin A (DZNep), an inhibitor of the histone methyltransferase EZH2, induces apoptosis and reduces cell migration in chondrosarcoma cells. PLoS ONE.

[5] Aury-Landas J., Girard N., Lhuissier E., Adouane D., Delépée R., Boumediene K., Baugé C. (2019). The Antitumoral Effect of the S-Adenosylhomocysteine Hydrolase Inhibitor, 3-Deazaneplanocin A, is Independent of EZH2 but is Correlated with EGFR Downregulation in Chondrosarcomas. Cell. Physiol. Biochem..

[6] Glazer R.I., Knode M.C., Tseng C.K., Haines D.R., Marquez V.E. (1986). 3-Deazaneplanocin A: A new inhibitor of S-adenosylhomocysteine synthesis and its effects in human colon carcinoma cells. Biochem. Pharmacol..

[7] Miranda T.B., Cortez C.C., Yoo C.B., Liang G., Abe M., Kelly T.K., Marquez V.E., Jones P.A. (2009). DZNep is a global histone methylation inhibitor that reactivates developmental genes not silenced by DNA methylation. Mol. Cancer Ther..

[8] Tan J., Yang X., Zhuang L., Jiang X., Chen W., Lee P.L., Karuturi R.K.M., Tan P.B.O., Liu E.T., Yu Q. (2007). Pharmacologic disruption of Polycomb-repressive complex 2-mediated gene repression selectively induces apoptosis in cancer cells. Genes Dev..

[9] Cheng L.L., Itahana Y., Lei Z.D., Chia N.-Y., Wu Y., Yu Y., Zhang S.L., Thike A.A., Pandey A., Rozen S. (2012). TP53 Genomic Status Regulates Sensitivity of Gastric Cancer Cells to the Histone Methylation Inhibitor 3-Deazaneplanocin A (DZNep). Clin. Cancer Res..

[10] Xie Z., Bi C., Cheong L.L., Liu S.C., Huang G., Zhou J., Yu Q., Chen C.-S., Chng W.J. (2011). Determinants of Sensitivity to DZNep Induced Apoptosis in Multiple Myeloma Cells. PLoS ONE.

[11] Ciarapica R., Carcarino E., Adesso L., De Salvo M., Bracaglia G., Leoncini P.P., Dall’agnese A., Verginelli F., Milano G.M., Boldrini R. (2014). Pharmacological inhibition of EZH2 as a promising differentiation therapy in embryonal RMS. BMC Cancer.

[12] Li Z., Wang Y., Qiu J., Li Q., Yuan C., Zhang W., Wang D., Ye J., Jiang H., Yang J. (2013). The polycomb group protein EZH2 is a novel therapeutic target in tongue cancer. Oncotarget.

[13] Crea F., Hurt E.M., Mathews L.A., Cabarcas S.M., Sun L., Marquez V.E., Danesi R., Farrar W.L. (2011). Pharmacologic disruption of Polycomb Repressive Complex 2 inhibits tumorigenicity and tumor progression in prostate cancer. Mol. Cancer.

[14] Liu L., Xu Z., Zhong L., Wang H., Jiang S., Long Q., Xu J., Guo J. (2016). Enhancer of zeste homolog 2 (EZH2) promotes tumour cell migration and invasion via epigenetic repression of E-cadherin in renal cell carcinoma. BJU Int..

[15] Tellez C.S., Picchi M.A., Juri D., Do K., Desai D.H., Amin S.G., Hutt J.A., Filipczak P.T., Belinsky S.A. (2021). Chromatin remodeling by the histone methyltransferase EZH2 drives lung pre-malignancy and is a target for cancer prevention. Clin. Epigenetics.

[16] Lhuissier E., Aury-Landas J., Bouet V., Bazille C., Repesse Y., Freret T., Boumédiene K., Baugé C., Lhuissier E., Aury-Landas J. (2018). Evaluation of the impact of S-adenosylmethionine-dependent methyltransferase inhibitor, 3-deazaneplanocin A, on tissue injury and cognitive function in mice. Oncotarget.

[17] Bray M., Driscoll J., Huggins J.W. (2000). Treatment of lethal Ebola virus infection in mice with a single dose of an S-adenosyl-L-homocysteine hydrolase inhibitor. Antiviral Res..

[18] Hung S.W., Mody H., Marrache S., Bhutia Y.D., Davis F., Cho J.H., Zastre J., Dhar S., Chu C.K., Govindarajan R. (2013). Pharmacological Reversal of Histone Methylation Presensitizes Pancreatic Cancer Cells to Nucleoside Drugs: In Vitro Optimization and Novel Nanoparticle Delivery Studies. PLoS ONE.

[19] Ni J., Hou X., Wang X., Shi Y., Xu L., Zheng X., Liu N., Qiu A., Zhuang S. (2019). 3-deazaneplanocin A protects against cisplatin-induced renal tubular cell apoptosis and acute kidney injury by restoration of E-cadherin expression. Cell Death Dis..

[20] Guo L., Lee Y.-T., Zhou Y., Huang Y. (2021). Targeting epigenetic regulatory machinery to overcome cancer therapy resistance. Semin. Cancer Biol..

[21] Jagasia A.A., Block J.A., Diaz M.O., Nobori T., Gitelis S., Inerot S.E., Iyer A.P. (1996). Partial deletions of the CDKN2 and MTS2 putative tumor suppressor genes in a myxoid chondrosarcoma. Cancer Lett..

[22] Scully S.P., Berend K.R., Toth A., Qi W.N., Qi Z., Block J.A. (2000). Marshall Urist Award. Interstitial collagenase gene expression correlates with in vitro invasion in human chondrosarcoma. Clin. Orthop..

[23] Duval E., Bigot N., Hervieu M., Kou I., Leclercq S., Galéra P., Boumediene K., Baugé C. (2011). Asporin Expression Is Highly Regulated in Human Chondrocytes. Mol. Med..

[24] Avan A., Crea F., Paolicchi E., Funel N., Galvani E., Marquez V.E., Honeywell R.J., Danesi R., Peters G.J., Giovannetti E. (2012). Molecular mechanisms involved in the synergistic interaction of the EZH2 inhibitor 3-deazaneplanocin A with gemcitabine in pancreatic cancer cells. Mol. Cancer Ther..

[25] Nakagawa S., Sakamoto Y., Okabe H., Hayashi H., Hashimoto D., Yokoyama N., Tokunaga R., Sakamoto K., Kuroki H., Mima K. (2014). Epigenetic therapy with the histone methyltransferase EZH2 inhibitor 3-deazaneplanocin A inhibits the growth of cholangiocarcinoma cells. Oncol. Rep..

[26] Unland R., Borchardt C., Clemens D., Kool M., Dirksen U., Frühwald M.C. (2015). Analysis of the antiproliferative effects of 3-deazaneoplanocin A in combination with standard anticancer agents in rhabdoid tumor cell lines. Anticancer Drugs.

