# Supplementary material for: Co-Treatment with the Epigenetic Drug, 3-Deazaneplanocin A (DZNep) and Cisplatin after DZNep Priming Enhances the Response to Platinum-Based Therapy in Chondrosarcomas
Source: Cancers (Basel). 2021 Sep 16;13(18):4648. doi: 10.3390/cancers13184648 (PMC8472299; doi:10.3390/cancers13184648)
Supplement: Supplementary file 1 [file cancers-13-04648-s001.zip › cancers-1280616-Table S1.pdf]

Table S1. Tumoral volumes of chondrosarcoma xenografts during priming and treatment periods. Volumes in mm^3

Mice	Group	Priming period (days)									Treatment period (days)						
		**0**	**4**	**7**	**11**	**14**	**18**	**21**	**25**	**28**	**32**	**35**	**39**	**42**	**46**	**49**	**53**
Animal C1	Control	207	252	293	353	348	335	443	508	691	908	1369	1901	3064	5122	5825	6936
Animal C2	Control	99	97	112	170	243	179	274	298	433	572	944	1165	1895	2805	3933	5051
Animal C3	Control	120	114	177	242	350	413	663	854	1044	1235	1706	2003	2976	3792	4824	5398
Animal C4	Control	90	103	90	130	98	97	103	108	122	151	234	359	481	763	1069	1340
Animal Cis1	Cisplatin	110	103	118	129	196	185	164	213	501	602	787	1083	1424	1975	2560	2910
Animal Cis2	Cisplatin	134	132	120	128	164	154	190	228	298	357	449	529	809	1002	1422	1313
Animal Cis3	Cisplatin	116	107	138	205	262	306	587	584	517	814	993	1114	1553	1940	2827	3689
Animal Cis4	Cisplatin	114	238	273	339	315	405	524	697	761	928	1474	2362	2958	4058	5895	5856
Animal Cis5	Cisplatin	120	126	126	118	155	273	347	641	891	1686	2135	3168	3172	4403	4649	6270
Animal Cis6	Cisplatin	98	107	115	117	151	168	169	243	358	489	637	817	1063	1357	1822	2483
Animal Cis7	Cisplatin	133	117	131	86	118	134	227	343	555	737	1125	1393	1928	2688	3355	4253
Animal DZ1	DZNep	92	96	103	87	138	86	143	135	181	218	290	350	459	633	998	1365
Animal DZ2	DZNep	154	92	66	99	173	130	281	359	530	519	633	745	1311	2197	3069	4030
Animal DZ3	DZNep	121	125	128	163	221	289	338	535	686	1019	2039	1852	1909	2299	3034	3326
Animal DZ4	DZNep	110	160	175	168	156	187	234	359	497	500	622	861	1043	1426	1755	2268
Animal DZ5	DZNep	147	74	116	160	167	197	279	309	352	362	395	421	654	1175	1395	1809
Animal DZ6	DZNep	120	135	127	126	201	247	314	562	694	1103	1454	2168	3043	3714	4424	5950

Animal DZ7	DZNep	109	113	103	105	111	104	87	96	78	108	133	157	255	251	342	473
Animal DZ-cis1	DZNep + Cisplatin	149	129	151	138	154	207	248	296	540	554	248	368	595	289	482	601
Animal DZ-cis2	DZNep + Cisplatin	99	77	88	119	115	126	126	159	208	262	326	363	411	627	589	791
Animal DZ-cis3	DZNep + Cisplatin	121	148	135	168	172	181	295	383	495	899	881	1033	1183	1244	1431	2170
Animal DZ-cis4	DZNep + Cisplatin	114	115	165	230	253	368	536	675	987	1512	1747	2597	3241	3851	4613	6079
Animal DZ-cis5	DZNep + Cisplatin	113	144	160	187	248	313	352	388	445	564	749	891	1055	1157	1281	1553
Animal DZ-cis6	DZNep + Cisplatin	166	260	352	425	472	696	697	1068	1256	1585	1821	2115	2313	3713	3445	5148
Animal DZ-cis7	DZNep + Cisplatin	114	124	78	103	102	111	125	132	195	283	423	587	718	906	1203	1757
