# Supplementary figures and images for: Co-Treatment with the Epigenetic Drug, 3-Deazaneplanocin A (DZNep) and Cisplatin after DZNep Priming Enhances the Response to Platinum-Based Therapy in Chondrosarcomas
Source: Cancers (Basel). 2021 Sep 16;13(18):4648. doi: 10.3390/cancers13184648 (PMC8472299; doi:10.3390/cancers13184648)

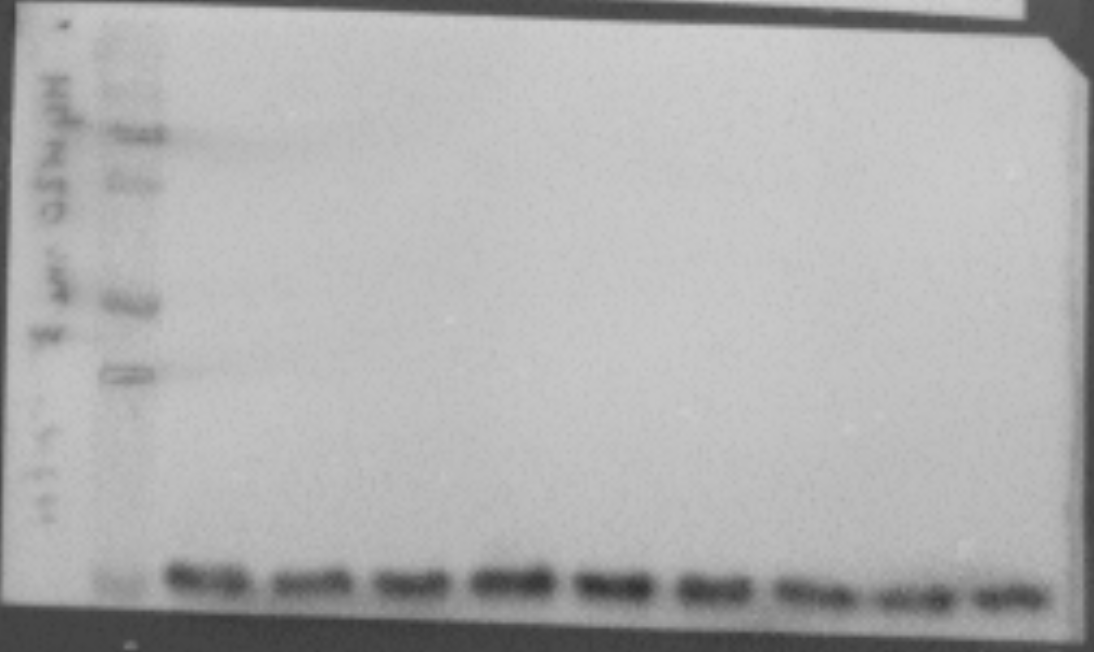

Supplement: Supplementary file 1 [file cancers-13-04648-s001.zip › cancers-1280616-File S1/H3-JJ.tif]

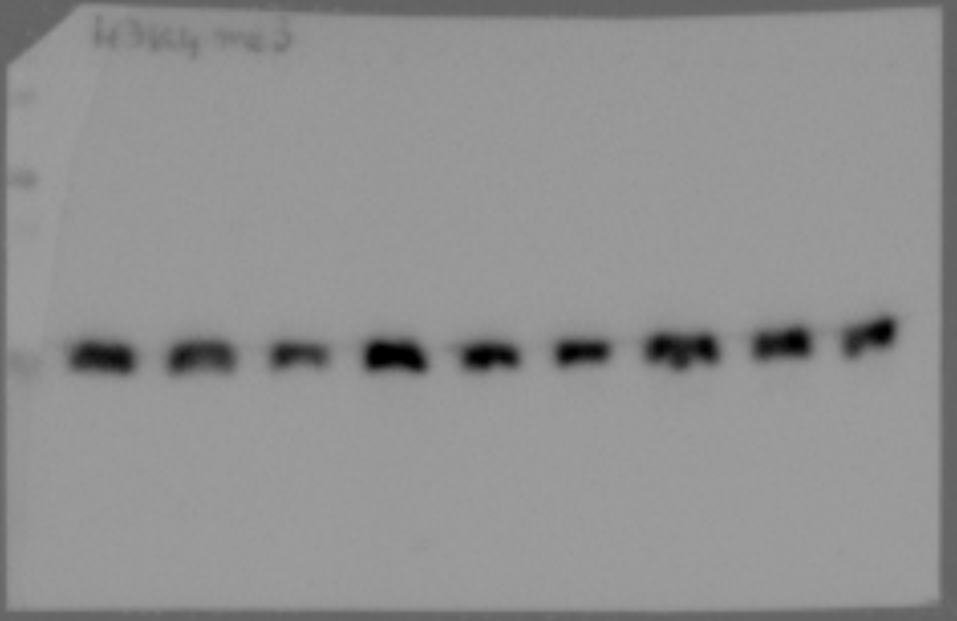

Supplement: Supplementary file 1 [file cancers-13-04648-s001.zip › cancers-1280616-File S1/H3-SW.tif]

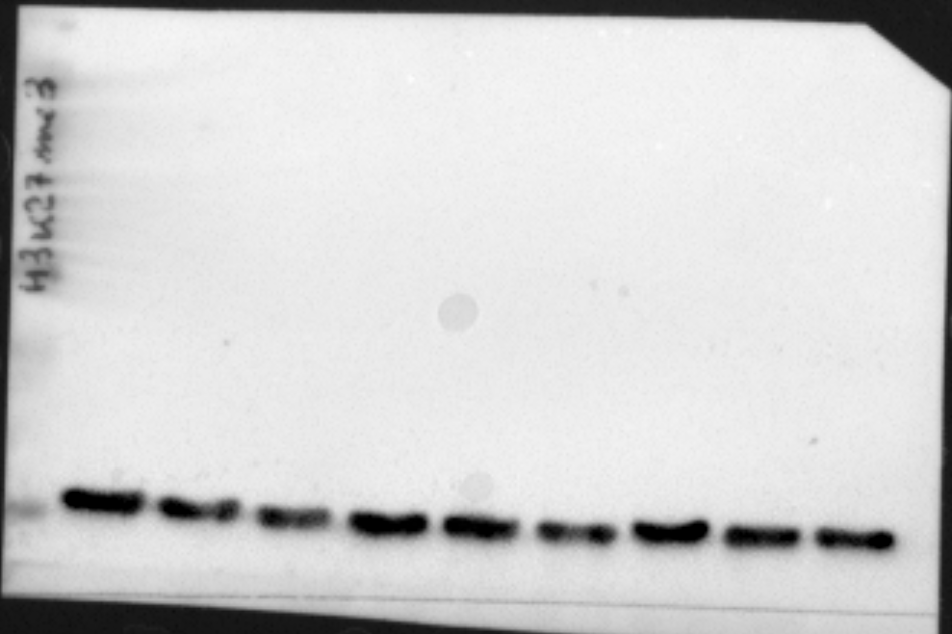

Supplement: Supplementary file 1 [file cancers-13-04648-s001.zip › cancers-1280616-File S1/H3K27me3-JJ.tif]

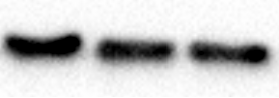

Supplement: Supplementary file 1 [file cancers-13-04648-s001.zip › cancers-1280616-File S1/H3K27me3-SW pour figure.tif]

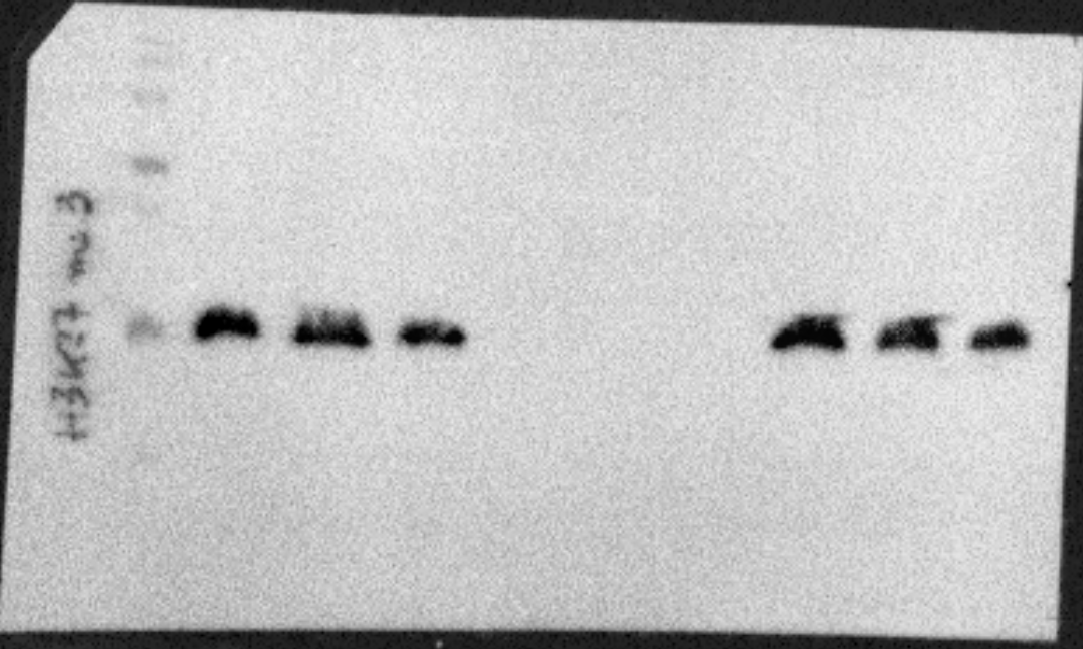

Supplement: Supplementary file 1 [file cancers-13-04648-s001.zip › cancers-1280616-File S1/H3K27me3-SW.tif]

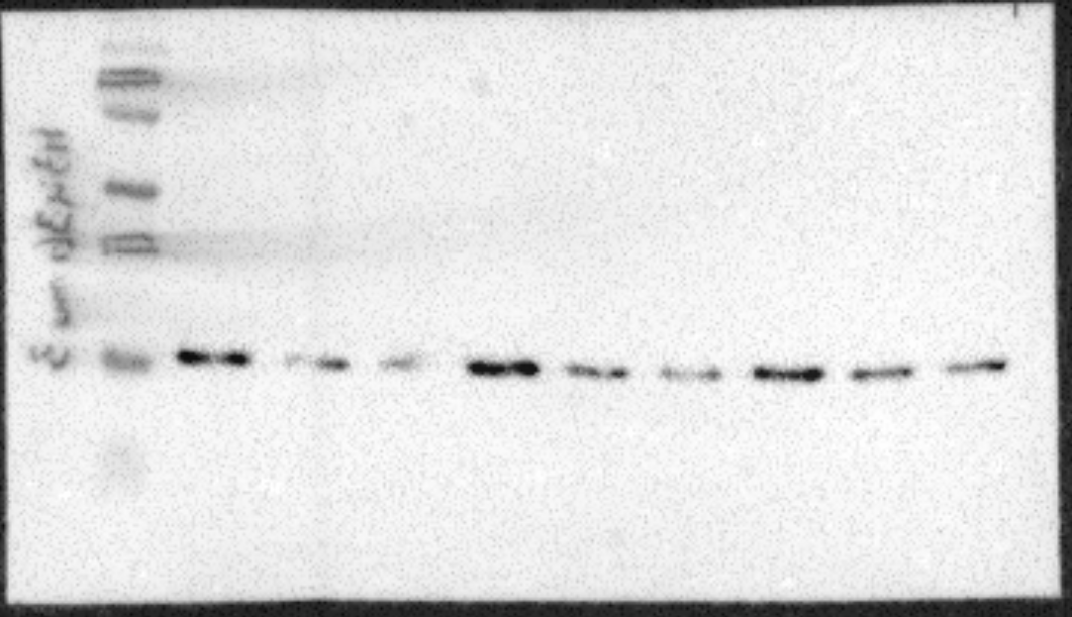

Supplement: Supplementary file 1 [file cancers-13-04648-s001.zip › cancers-1280616-File S1/H3K36me3-JJ.tif]

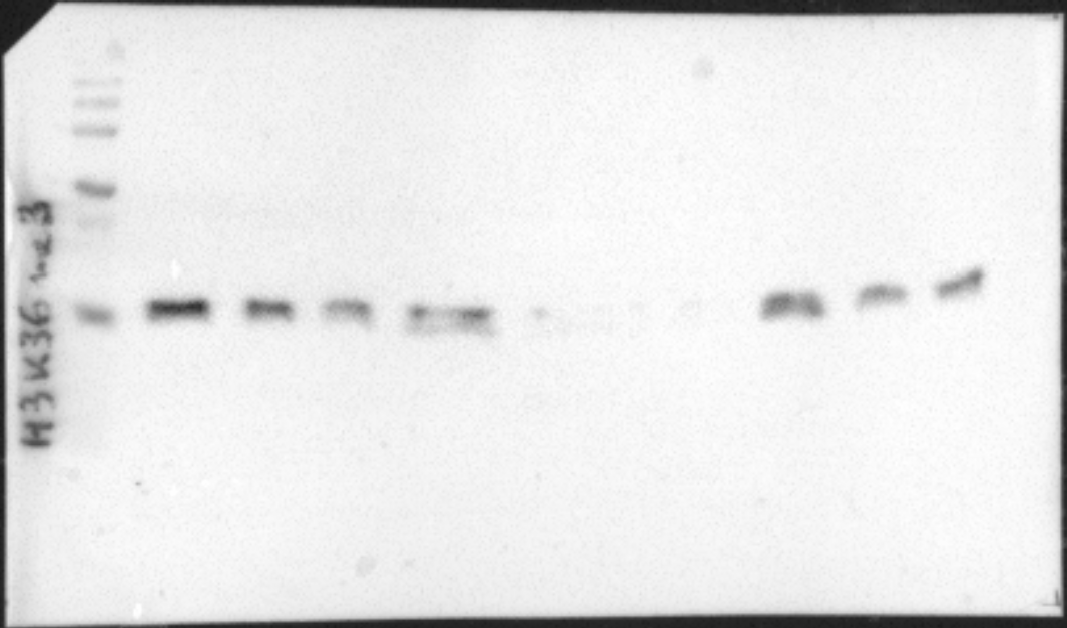

Supplement: Supplementary file 1 [file cancers-13-04648-s001.zip › cancers-1280616-File S1/H3K36me3-SW.tif]

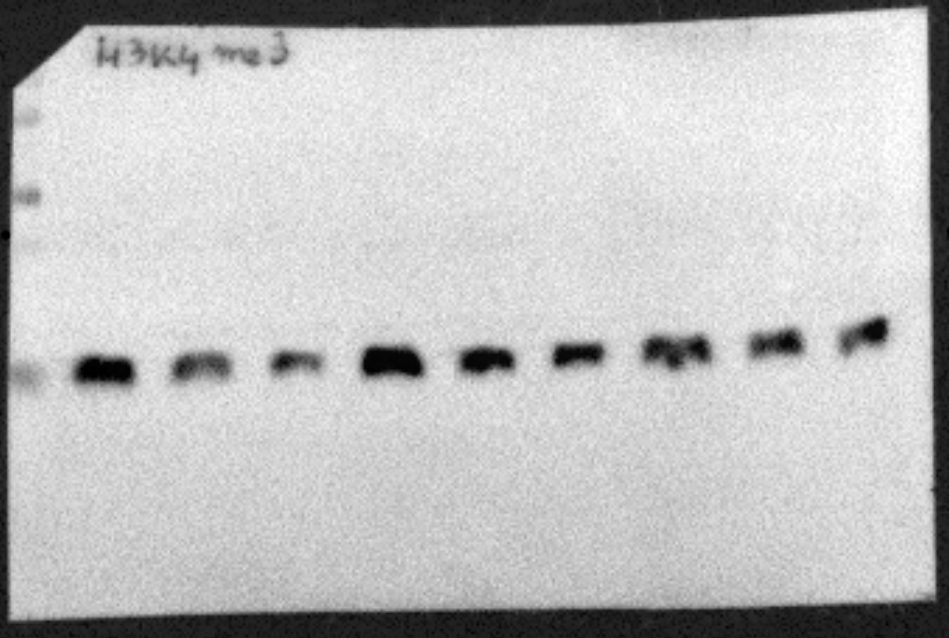

Supplement: Supplementary file 1 [file cancers-13-04648-s001.zip › cancers-1280616-File S1/H3K4me3-SW-JJ.tif]

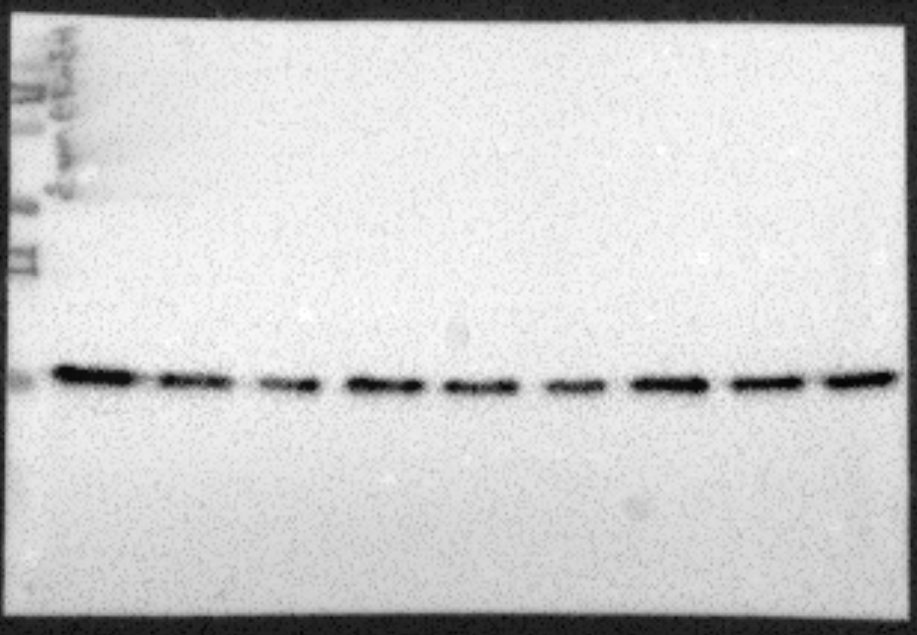

Supplement: Supplementary file 1 [file cancers-13-04648-s001.zip › cancers-1280616-File S1/H3K79me3-JJ.tif]

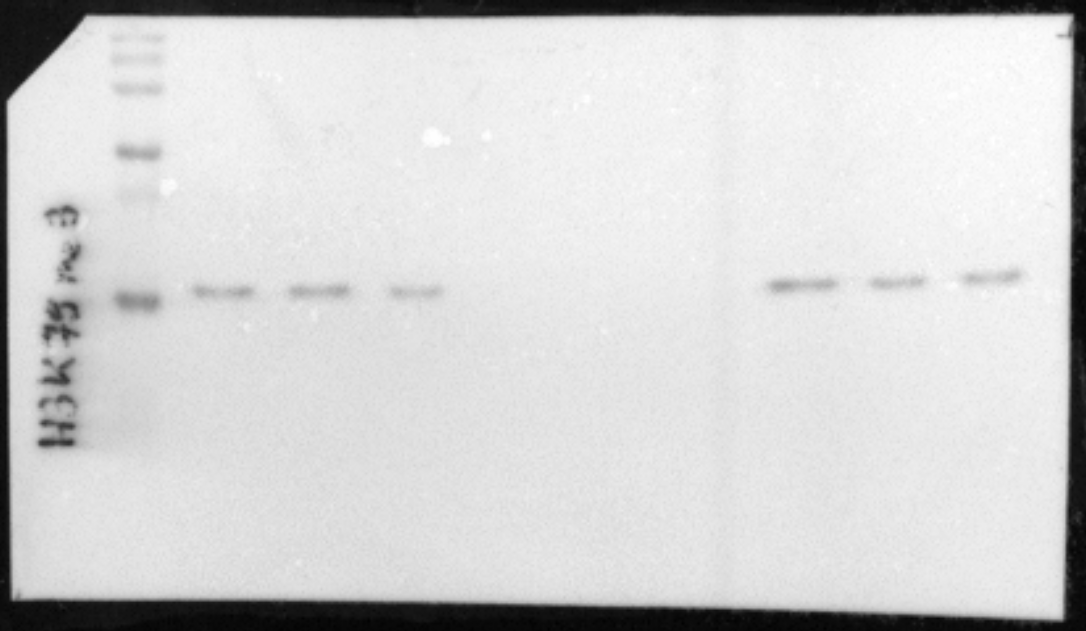

Supplement: Supplementary file 1 [file cancers-13-04648-s001.zip › cancers-1280616-File S1/H3K79me3-SW.tif]

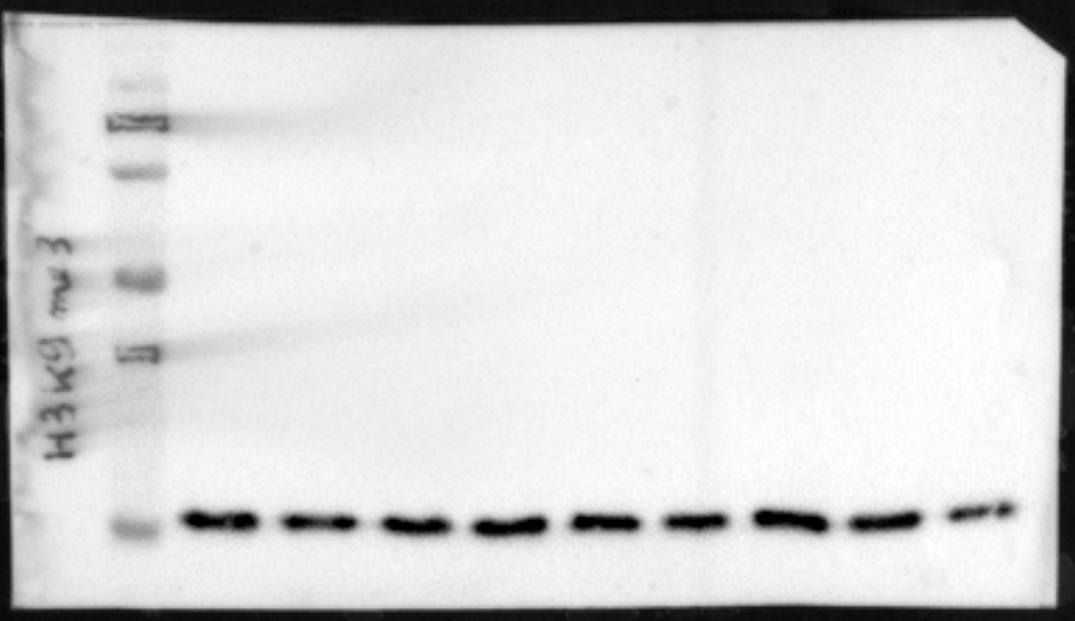

Supplement: Supplementary file 1 [file cancers-13-04648-s001.zip › cancers-1280616-File S1/H3K9me3-JJ.tif]

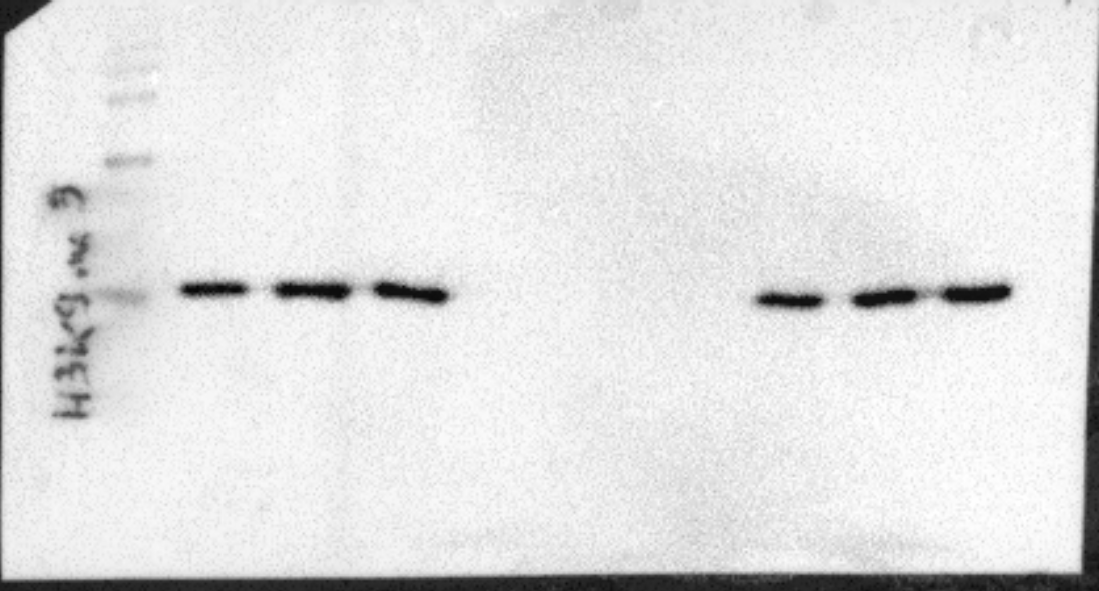

Supplement: Supplementary file 1 [file cancers-13-04648-s001.zip › cancers-1280616-File S1/H3K9me3-SW.tif]

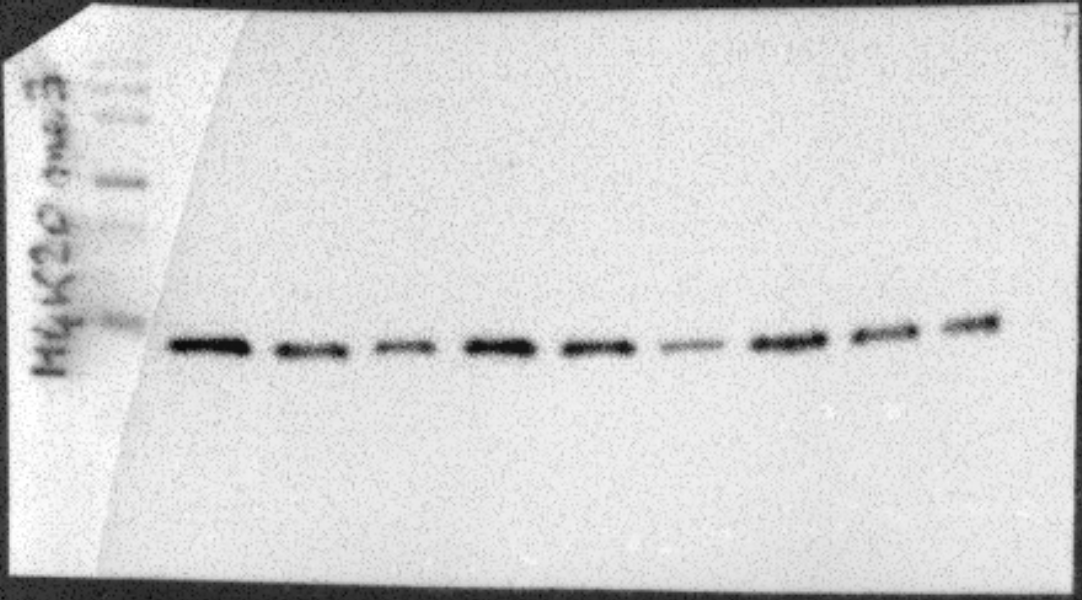

Supplement: Supplementary file 1 [file cancers-13-04648-s001.zip › cancers-1280616-File S1/H4K20me3-JJ.tif]

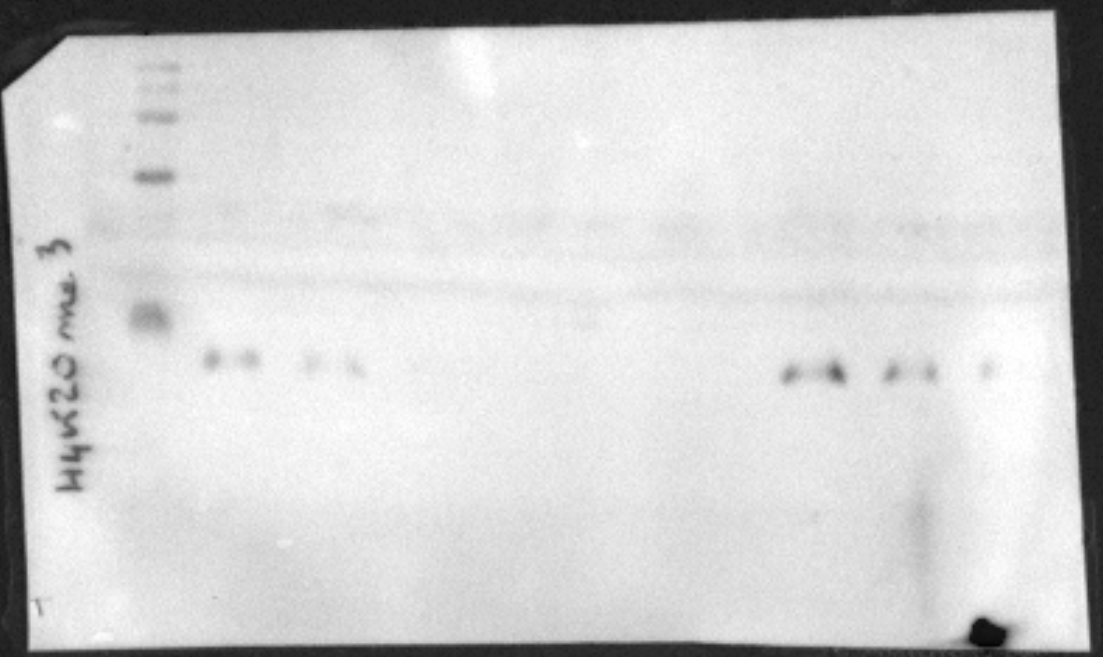

Supplement: Supplementary file 1 [file cancers-13-04648-s001.zip › cancers-1280616-File S1/H4K20me3-SW.tif]
